# RETRACTION: Effect of Intraoperative Abdominal Lavage Versus Suction Alone on Postoperative Wound Infection in Patients With Appendicitis: A Meta‐Analysis

**DOI:** 10.1111/iwj.70387

**Published:** 2025-03-21

## Abstract

**RETRACTION:** WuH. , ChenX. , RenY. , and YangF. , “Effect of Intraoperative Abdominal Lavage Versus Suction Alone on Postoperative Wound Infection in Patients With Appendicitis: A Meta‐Analysis,” International Wound Journal 21, no. 4 (2024): e14613, 10.1111/iwj.14613.38158647 PMC10961855

The above article, published online on 29 December 2023, in Wiley Online Library (http://onlinelibrary.wiley.com/), has been retracted by agreement between the journal Editor in Chief, Professor Keith Harding; and John Wiley & Sons Ltd. Following an investigation by the publisher, all parties have concluded that this article was accepted solely on the basis of a compromised peer review process. The editors have therefore decided to retract the article. The authors did not respond to our notice regarding the retraction.

**RETRACTION:** H. Wu , X. Chen , Y. Ren , and F. Yang , “Effect of Intraoperative Abdominal Lavage Versus Suction Alone on Postoperative Wound Infection in Patients With Appendicitis: A Meta‐Analysis,” International Wound Journal 21, no. 4 (2024): e14613, 10.1111/iwj.14613.38158647 PMC10961855

The above article, published online on 29 December 2023, in Wiley Online Library (http://onlinelibrary.wiley.com/), has been retracted by agreement between the journal Editor in Chief, Professor Keith Harding; and John Wiley & Sons Ltd. Following an investigation by the publisher, all parties have concluded that this article was accepted solely on the basis of a compromised peer review process. The editors have therefore decided to retract the article. The authors did not respond to our notice regarding the retraction.

